# A fully automated machine learning assisted pipeline to predict disease progression for early-stage hypertrophic cardiomyopathy from echocardiography

**DOI:** 10.3389/fcvm.2026.1875305

**Published:** 2026-08-03

**Authors:** Antoine Olivier, Auriane Riou, Thomas d’Humières, Maxime Touzot, Kévin Elgui, Benoît Sauty, Paul Trichelair, Valérie Ducret, Maria Telenczuk, Antoine Simon, Felix Balazard, Mariann Micsinai Balan, Arnaud Bastien, Amy Sehnert, Faiez Zannad, Philippe Gabriel Steg, Perry Blizard, Lauren Turvey-Haigh, Tomas Ripoll, William Bradlow, Philippe Charron

**Affiliations:** 1 Owkin France, Paris, France; 2 Physiology Department, FHU SENEC, Henri-Mondor Hospital, Assistance Publique-Hôpitaux de Paris, Créteil, France; 3 Bristol-Myers Squibb, Princeton, NJ, United States; 4 University of Lorraine, Nancy, France; 5 Université Paris-Cité, INSERM UMR1148 and AP-HP, Paris, France; 6 Department of Cardiology, University Hospitals Birmingham NHS Foundation Trust, Birmingham, United Kingdom; 7 Cardiology Department, Hospital Universitari Son Llàtzer, Research Institute of Health Sciences (IdISBa), Palma, Spain; 8 Department of Cardiology & Department of Genetics, INSERM UMRS-1166, ICAN Institute, Pitié-Salpêtrière Hospital, Sorbonne University, APHP, Paris, France

**Keywords:** echocardiography, hypertrophic cardiomyopathy, left atrioventricular coupling index, machine learning, risk stratification

## Abstract

**Introduction:**

Echocardiography is critical for the diagnosis and risk stratification of hypertrophic cardiomyopathy (HCM). However, its value to predict disease progression in pre-symptomatic HCM remains to be fully explored. This study aims to assess the prognostic value of echocardiography in pre-symptomatic HCM using a fully automated machine learning (ML) pipeline to predict disease progression.

**Methods:**

Echocardiographic data (B-mode acquisitions of apical 4-chamber sequences) from 260 NYHA I HCM patients, collected retrospectively from two centers, was used. An ML pipeline was built on the discovery cohort (N=212 patients) to predict disease progression, defined as a composite of NYHA class worsening and unplanned cardiovascular-related hospitalizations. A fully automated deep learning segmentation pipeline was used to delineate cardiac chambers in apical 4-chamber views and identify end-diastolic frames. Shape-based radiomic features extracted from these segmentations were used to train a survival model based on gradient-boosted trees. The ML model and a derived actionable echocardiographic marker were validated on an external cohort (N=48 patients).

**Results:**

The 3-year risk of disease progression was 12% in the discovery cohort and 15% in the validation cohort. The ML model achieved a C-index of 0.66 (95% CI [0.54, 0.77], nested cross-validation folds) in the discovery cohort and 0.67 (95% CI [0.46, 0.88], 100 bootstrapped samples) in the validation cohort. Following model interpretation, the left atrioventricular coupling index (area-derived LACI) at end-diastole was derived, and used as a risk score, achieving a C-index of 0.67 (95% CI [0.58, 0.77]) and 0.72 (95% CI [0.56, 0.88]) in the discovery and validation cohorts, respectively (100 bootstrapped samples). The high-risk group, with LACI>0.51, had a 3-year risk of disease progression of 19% (95% CI [13%, 36%]) compared to 8% (95% CI [5%, 15%]) for the low-risk group LACI ≤0.51 in the discovery cohort.

**Conclusion:**

A fully automated ML model identifies area-derived LACI at end-diastole as a robust feature associated with disease progression, providing improved risk stratification for pre-symptomatic HCM.

## 1 Introduction

Hypertrophic cardiomyopathy (HCM) is one of the most common inherited cardiac disorders [1, 2]. Although considered a monogenic disease targeting sarcomeric protein, the variability of patients’ genetic backgrounds and epigenetic mechanisms results in a large phenotypic variability, with highly singular natural histories (3, 4). While most patients tend to remain asymptomatic with a normal life expectancy, some will eventually develop cardiac complications such as atrial fibrillation (AF), sudden cardiac death (SCD) or heart failure (HF) (3, 5, 6).

Transthoracic echocardiography (TTE) is the cornerstone of screening and follow-up of HCM (3, 6), as it allows to assess left ventricular wall thickness, and features such as systolic anterior motion (SAM) of the mitral valve, left ventricular outflow tract (LVOT) obstruction and diastolic or systolic dysfunction. Cardiac magnetic resonance (CMR) imaging might complement echocardiography assessment for early diagnostic work-up and identification of additional prognostic markers such as late gadolinium enhancement (LGE) reflecting the extent of cardiac fibrosis. Since longitudinal CMR imaging evaluation cannot be easily performed in routine clinical practice, TTE remains the main and most accessible way to repeatedly assess cardiac function, morphology, remodeling and obstruction in HCM. During the last decade, several parameters have been associated with cardiac outcomes in HCM, either clinical symptoms, cardiac imaging parameters or other clinical parameters (3, 7). Notably, echocardiographic parameters related to the left atrium (LA) enlargement have been shown to predict major adverse cardiac events (8, 9) in HCM, including new onset of AF (10–12) and other cardiac and cerebrovascular adverse events (13).

Robust models have been developed to predict the risk of SCD or AF in HCM patients but few have focused on the progression of disease-related structural cardiac abnormalities or symptoms. A machine learning model including clinical symptoms and cardiac remodeling was recently proposed, and was successful in predicting heart failure occurrence over subsequent 10 years (14). However, most of these models or markers have been identified and validated in symptomatic patients (New York Heart association NYHA > I), and it remains unclear whether it also translates in less severe patients such as pre-symptomatic HCM patients.

In the present work, leveraging raw echocardiographic data from the predictorS Of cLinical outcomEs iN hypertrOphIc carDiomyopathy (SOLENOID) registry of pre-symptomatic patients (15), a fully automated machine learning (ML) model to predict disease progression for early-stage HCM patients was developed. Second the left atrioventricular coupling index (area-derived LACI) was derived, and its relevance validated, that can offer independent risk stratification for these patients.

## 2 Materials and methods

### 2.1 Study population

#### 2.1.1 Inclusion criteria

The SOLENOID registry is a real-world, retrospective observational study conducted across five academic centers in Europe and the US (15). Briefly, inclusion criteria were:

- Adult patients with confirmed or presumed sarcomeric HCM (e.g., following genetic testing, family history, CMR or echo-based testing) categorized as NYHA class I between 2010 and 2022 were enrolled. Genetic testing was not required for inclusion and was available only in a subset of patients.
- Availability of raw echocardiography data (at least one B-mode apical 4-chamber sequence).

Patients with non-sarcomeric HCM (3) such as amyloidosis, hemochromatosis, Fabry disease, left ventricular hypertrophy (LVH) in athletic heart syndrome or hypertensive disease or aortic valve stenosis were excluded.

#### 2.1.2 Data collection

Patients’ demographics and laboratory data were collected using medical informatics record systems.

Demographic variables comprised age and gender. Medical history encompassed a range of conditions, including history of AF, history of arterial hypertension, diabetes. Physical examination variables included body mass index (BMI), systolic and diastolic blood pressure, and NYHA class assessment. Treatments at baseline were also recorded, focusing on the use of medications such as beta-blockers (BB)- and non-dihydropyridine calcium channel blockers (CCB). Assessments from echocardiography included left ventricular ejection fraction (LVEF), maximal end-diastolic LV wall thickness, LAVi, left atrial area, left atrial diameter, velocity, E/e’ lateral ratio, LVOT gradient, and LV mass index. Laboratory tests included measurements of NT-proBNP. Additionally, results from HCM genetic panel testing were collected when available, including results for pathogenic variants in MYH7 and MYBPC3 genes.

Additionally, raw echocardiography data were available in 2 centers: University Hospitals Birmingham (UHB, UK cohort), used as the discovery cohort, and the Fundació Institut d’Investigació Sanitària Illes Balears (IDISBA, Spanish cohort), used for external validation. B-mode apical 4-chamber echocardiographic sequences were collected for 212 and 48 patients from the UK and Spanish cohort, respectively.

#### 2.1.3 Composite endpoint

The study’s outcome was a composite of NYHA class worsening and unplanned cardiovascular hospitalization events (SCD, heart failure, diagnosis of AF, stroke), as described in the SOLENOID registry (15).

### 2.2 Prognostic model development

**Figure 1:**
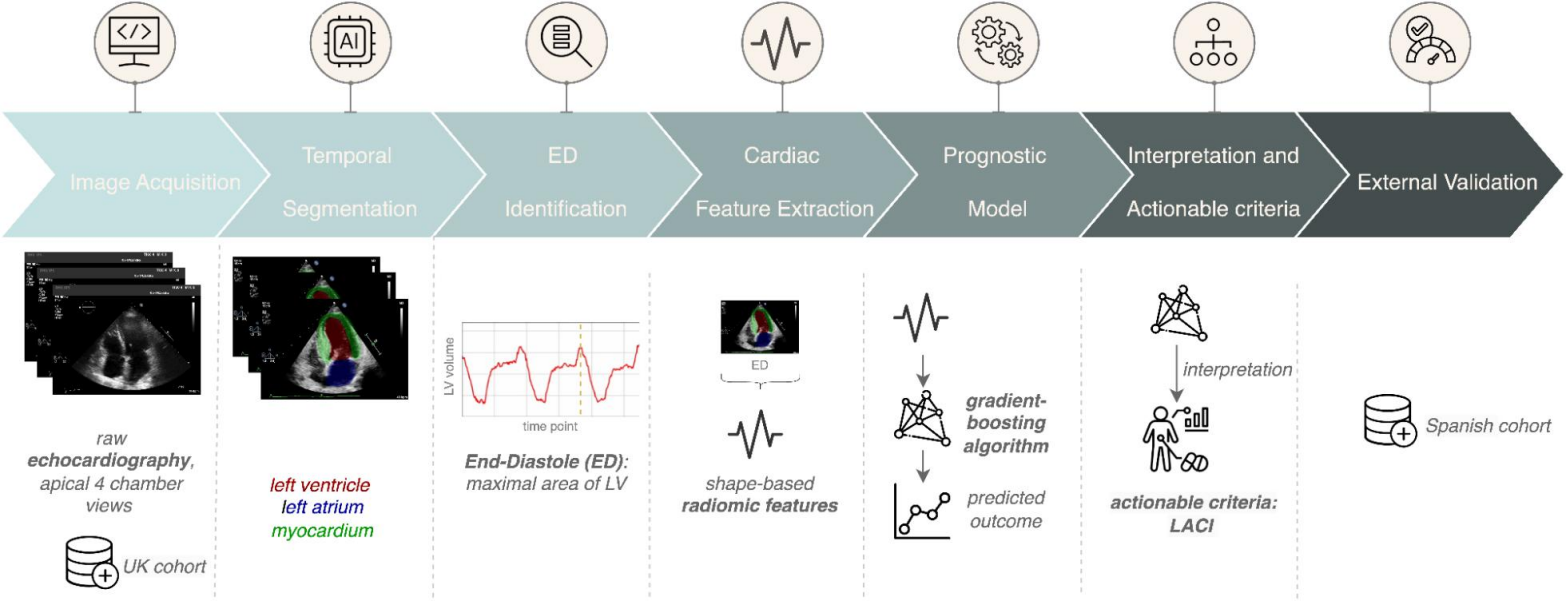
Sequential steps for prognostic model development in early-stage hypertrophic cardiomyopathy (HCM) using echocardiography. ED, end-diastole; LV, left ventricle; LACI, area-derived left atrioventricular coupling index.

Figure 1 illustrates the main sequential steps implemented in the study. A more detailed version of each step is given in Supplementary Material.

#### 2.2.1 Cardiac segmentation and end-diastole identification

The first step consisted of automatically segmenting the cardiac structures in echocardiographic images. This was performed using a supervised Deep Learning segmentation model based on the U-net (16) architecture. The model was trained using the publicly available “Cardiac Acquisitions for Multi-structure Ultrasound Segmentation” (CAMUS) dataset (17), which contains labeled examples of cardiac structures. For each new B-mode apical 4 chamber view, the trained model produced a segmentation mask that delineated the three main structures on the left side of the heart: the myocardium, left ventricle and left atrium. The temporal evolution of the left ventricle area was then used to automatically identify an end-diastole (ED) frame for each patient, by selecting the maximal measured LV area across the sequence. Finally, to assess the reliability of the segmentation, the agreement between the automatically extracted LA area and measurements obtained by a clinician was evaluated using both Bland-Altman and correlation analyses.

#### 2.2.2 Feature extraction and model training

For each patient, and based on the previous segmentation step, radiomics were extracted for each structure of the ED frame. To prioritize the use of reproducible and interpretable features, the analysis was exclusively focused on shape features, describing the geometry of each structure. A supervised survival model was then trained to predict disease progression from extracted radiomics. Specifically, Gradient Boosting Trees were used, optimized to maximize the log likelihood for the Accelerated Failure Time (AFT) (18) which is well suited for modeling time-to-event problems. To avoid overfitting, and in order to accurately estimate the concordance index (C-index), hyperparameters were chosen on the discovery cohort using a nested cross-validation. This approach allowed for a fair and unbiased estimation of the model’s predictive accuracy. The uncertainty of the cross-validated C-index estimate was quantified using a corrected resampled t-test accounting for the dependence between repeated cross-validation estimates, following Nadeau and Bengio (19). Lift curves (20) were also computed to evaluate how effectively the model could separate patients into different risk groups at a specific time point, thereby providing a visual measure of its stratification capability.

#### 2.2.3 Model validation

The model was validated on an external validation cohort. The generalization performance of the model was similarly assessed using the C-index and lift curves. Bootstrapping techniques were employed (n=100), which allowed to derive 95% confidence intervals.

Calibration of the prognostic model was assessed in the external validation cohort by comparing 3-year predicted probabilities of disease progression with observed event rates estimated using Kaplan-Meier estimates at 3 years. Predicted probabilities were derived from the survival distribution generated by the AFT model. Uncertainty in the Expected/Observed (E/O) ratio was quantified using bootstrap resampling (n=500). The distribution of log(E/O) was used to construct confidence intervals, which were exponentiated to obtain bounds on the original scale. In addition, patients were stratified into tertiles of predicted risk to visually compare predicted and observed event rates across risk groups.

#### 2.2.4 Model interpretation

To interpret the prognostic model and assess the influence of various features on risk prediction, Shapley (SHAP) values (21) were computed. This method quantifies, for each feature and for each patient, the extent to which that feature contributes to increase or decrease the predicted risk of disease progression. Additionally, univariate analyses were conducted and allowed, for each individual input feature of the prognostic model, to assess its association with the composite endpoint.

### 2.3 Echocardiographic marker development and evaluation

#### 2.3.1 Definition and derivation of an echocardiographic marker

The contribution of each individual feature to the risk predicted by the model was assessed. By looking at the most prognostic features, and in line with prior knowledge, the echocardiographic marker used in this study is the left atrioventricular coupling index (area-derived LACI). It is measured at end-diastole (ED) and defined as the ratio between the left atrial end-diastolic area (LAEDA) and the left ventricular end-diastolic area (LVEDA), both obtained from the automated segmentation pipeline: $\frac{\mathrm{LAEDA}}{\mathrm{LVEDA}}$. This differs from the original volumetric LACI described in the literature, which is based on left atrial and left ventricular volumes measured by cardiac imaging (22, 23). To avoid ambiguity, we refer to this metric as the area-derived LACI throughout the remainder of the manuscript. This index was used directly as a risk score, or to stratify the population.

#### 2.3.2 Assessment of echocardiographic marker performance

Three complementary approaches were used.

1. The performance of the echocardiographic marker was assessed using a bootstrapped C-index (with n=100 repetitions) on both the discovery and validation cohorts. 95% confidence intervals were derived assuming a normal distribution of the bootstrap C-index estimates.
2. For each center, a univariate analysis was conducted to assess the association between the echocardiographic marker and classical prognostic echocardiographic biomarkers (including maximal end-diastolic left ventricular wall thickness, E/e’ lateral ratio, LAVi, and LA or LV areas) and the endpoint. Each feature was standardized using the Yeo-Johnson transformation (24). The Yeo-Johnson transform is a monotonic transform allowing to make a distribution more gaussian-like, better suited than z-normalization for asymmetric distributions. Univariate Cox regression was then applied to estimate the association between each transformed variable and the outcome, adjusting for age. The log hazard ratio (logHR) and associated p-values for each individual predictor variable were reported. Missing values for each variable were excluded from the univariate analysis. *P*-values were adjusted for false discovery rate using the Benjamini/Hochberg (BH) procedure (25, 26) at a 5% level, with the total number of features analyzed serving as the number of tests.
3. Finally, the derived echocardiographic marker was used to stratify the population into two distinct groups regarding the risk of disease progression (high vs low risk). The SOLENOID study highlighted a 3-year incidence rate for the composite endpoint close to 30%. Therefore, a threshold, determined from data at the UK cohort, identified the top 30% of patients with highest area-derived LACI values, ensuring that the high-risk group was aligned with the study’s overall risk profile. This threshold was then applied to the Spanish cohort. In both centers, patients with area-derived LACI values exceeding the threshold were classified as high-risk, while those at or below the threshold were considered low-risk. Survival distributions between these groups were compared using log-rank tests. To assess the robustness of the threshold, sensitivity analyses were performed using alternative cutoffs (25% and 35% of the population), as well as an additional analysis using a 15% cutoff aligned with the observed event rate.

## 3 Ethical statement

The study was sponsored by Bristol-Myers Squibb. The study was approved by the institutional ethics committee or Institutional Review Board (IRB) at each site. For all study participants, an informed consent form with nonopposition principle for the reuse of their health data for research purposes was communicated to the patient at the time of admission in each of the study centers. All patient data were anonymized to ensure privacy and confidentiality. The research team adhered to all relevant guidelines for human subjects research.

## 4 Results

### 4.1 Cohort characterization

From the original SOLENOID registry, 260 (25%) patients had available raw echo images at baseline and were included in the present study. The cohort was divided into a discovery cohort (UK, N=212) and the validation cohort (Spain, N=48). Table 1 summarizes the baseline characteristics and clinical information, as well as echocardiographic (from reports), laboratory and genetic parameters. Echocardiographic parameters identified and estimated by the model segmentation are also summarized in Table 1. Briefly, the discovery cohort included HCM patients with a low risk profile. The mean age was 52±16 years. Only 9% of patients had a history of AF, and 7% had type 2 diabetes (T2D). Among patients that had undergone genetic testing, MYH7-mutated patients represented 9% while MYBPC3-mutated patients represented 32%. The mean systolic blood pressure (SBP) was 141±19 mmHg and the mean diastolic blood pressure (DBP) 84±11 mmHg. Baseline NT-ProBNP was available for 146 patients (69%) of the population, and the mean value was 527±741 pg/ml. 40% of the patients were on BBs and 3% on non-dihydropyridine Calcium channel blockers (CCB). The mean LVEF was 66±7%, the mean LAVi value was $34\pm 13\;{\mathrm{mL}/m}^{2}$. Few patients (12%) had obstruction detected on echocardiography. The echocardiographic parameters reported in Table 1 were extracted from the clinical reports. The baseline characteristics were similar in the validation cohort, except for non-dihydropyridine CCB treatment at baseline, systolic blood pressure and left ventricular area extracted by the segmentation pipeline.

**Table 1:** Baseline characteristics for the discovery and validation cohorts.

	UK (N=212)		SPAIN (N=48)		*p* values
Variable	Observed (Missing)	Desc.	Observed (Missing)	Desc.	
Demographics					
Gender–Male	212 (0)	83%	48 (0)	79%	p=0.927
Age–Years	212 (0)	52±16	48 (0)	55±17	p=0.514
NYHA class					
NYHA class I	212 (0)	100%	48 (0)	100%	na
Medical history					
History of AF	212 (0)	9%	48 (0)	0%	na
Arterial Hypertension	212 (0)	37%	48 (0)	31%	p=0.784
Type 2 diabetes	212 (0)	7%	48 (0)	0%	na
Physical examination					
BMI (kg/m${}^{2}$)	204 (8)	28.6±4.7	48 (0)	27.8±4.7	p=0.619
Systolic Blood Pressure (mmHg)	207 (5)	141±19	24 (24)	129±15	p=0.018
Diastolic Blood Pressure (mmHg)	207 (5)	84±11	24 (24)	79±11	p=0.182
Treatment at baseline					
Beta-blockers	212 (0)	40%	48 (0)	33%	p=0.704
Non-dihydropyridine CCB	212 (0)	3%	48 (0)	15%	p=0.035
HCM genotyping					
MYH7 mutated	182 (30)	9%	19 (29)	11%	p=1.000
MYBPC3 mutated	185 (27)	32%	19 (29)	32%	p=1.000
Echocardiography (Reports)					
LVEF (%)	121 (91)	66±7	7 (41)	74±12	p=0.352
Maximal end-diastolic LV wall thickness (mm)	130 (82)	18±4.8	0 (48)	–	na
LAVi (mL/$m^{2}$)	151 (61)	34±13	4 (44)	33±6	p=0.981
Left atrial area (cm${}^{2}$)	46 (166)	23±5.6	0 (48)	–	na
Left atrial diameter (mm)	42 (170)	41±7.2	10 (38)	45±6	p=0.182
E/e’ lateral ratio	136 (76)	9.4±3.1	13 (35)	9±3	p=1.000
LVOT gradient (mmHg) (at rest or at Valsalva)	149 (63)	17.4±31	4 (44)	38±37	p=0.619
LVOT gradient >30 mmHg	149 (63)	12%	4 (44)	50%	p=0.352
LV mass index (${g/m}^{2}$)	42 (170)	123±44	1 (47)	–	na
Echocardiography (segmentation pipeline)					
Left atrial area (${\mathrm{cm}}^{2}$)	212 (0)	14.5±6.0	48 (0)	12.9±4.6	p=0.225
Left ventricular area (${\mathrm{cm}}^{2}$)	212 (0)	33.2±7.0	48 (0)	28.7±8.4	p=0.018
Laboratory test					
NT-proBNP (pg/ml)	146 (66)	527±741	2 (46)	279±286	p=0.671

When available, echocardiographic parameters were extracted from reports of exams within a three-month window around the acquisition date of the ultrasound images used in this study. When floating point values are reported, it corresponds to the mean (±std) value over all available data (excluding missing values). When percentages are reported, it corresponds to the percentage of patients expressing a characteristic, over all available data (excluding missing values). Statistical differences between the two cohorts are assessed with a Welch’s test for continuous variables, and with a chi-square contingency test for categorical variables.

*p*-values are corrected for multiple testing with the Benjamini-Hochberg procedure.

HCM, Hypertrophic cardiomyopathy; AF, atrial fibrillation; NYHA, New York Heart Association; BMI, body mass index; CCB, Calcium channel blockers; LVEF, left ventricular ejection fraction; LAVi, left atrial volume index; LV, left ventricle; LVOT, left ventricular outflow tract; NT-proBNP, N-terminal pro b-type natriuretic peptide; Desc., description, mean (standard deviation) for continuous variables and percentage for binary variables.

### 4.2 Main outcome

The 3-year cumulative risk of disease progression for the composite endpoint was 12% in the discovery cohort and 15% in the validation cohort, assessed with Kaplan-Meier analyses Supplementary Figure S1, with 21 and 6 events occurring within 3 years in the discovery and validation cohorts respectively.

### 4.3 Prognostic model performance

#### 4.3.1 Internal validation on the UK cohort

##### 4.3.1.1 Segmentation model

**Figure 2:**
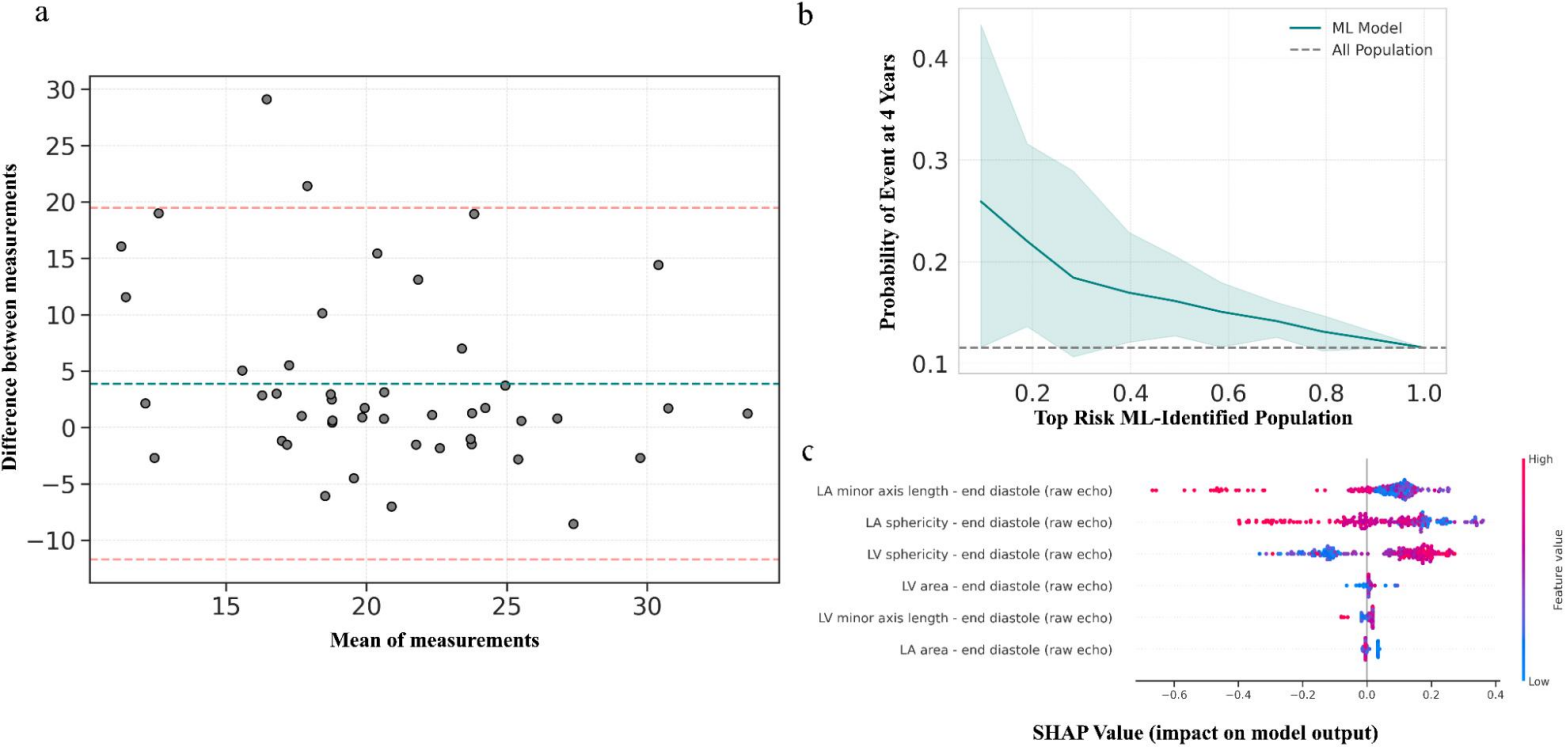
**(a)** Bland-Altman plot comparing automatically extracted LA area at ES with clinician measurements for 46 patients. The mean of the two measurements is plotted against their difference. The bias (mean difference) and 95% limits of agreement (±1.96 SD) are shown as dashed lines. **(b)** Lift curve at 4 years for the prognostic model in the discovery cohort. 50 splits were performed, using 75% of the data for training and 25% for validation. For each split, a model was trained on the training set using the hyperparameter optimization pipeline, and a lift curve was estimated based on the predictions on the validation set. The main curve is derived from the average across all curves, while the confidence intervals are based on the 0.1 and 0.9 quantiles of the predicted values. **(c)** Beeswarm plot of the SHAP (Shapley) values for the prognostic model trained on the full discovery cohort, showing some top prognostic features. Each instance is represented by a single dot on each feature row. The x position of the dot is determined by the SHAP value of that feature, and dots pile up along each feature row to show density. The features are ordered from top to bottom by their maximum contribution to the model. Color is used to display the original value of a feature going from blue (low value) to red (high value). All features are measured at ED. LA, left atrium; ES, end-systole; ED, end-diastole; SD, standard deviation; LV, left ventricle.

Echocardiographic parameters identified and estimated by the model segmentation are also summarized in Table 1. The agreement between automatically extracted LA area from raw images and clinician measurements (from echocardiographic reports) was assessed using a Bland-Altman plot (Figure 2a) for 46 patients. The correlation between the two measurements is shown in Supplementary Figure S7 (Pearson’s r=0.26, p=0.077).

##### 4.3.1.2 ML model performance

Using a nested cross-validation strategy, the ML model, trained on raw echocardiographic data from 212 patients in the UK cohort, reached a C-index of 0.66 (95% CI [0.54, 0.77]). The lift curve estimated in the UK cohort, shown in Figure 2b, highlights the model’s effectiveness in ranking individuals by risk at a given timepoint, compared to random selection.

##### 4.3.1.3 ML model interpretability

The SHAP values, presented in Figure 2c, provide insights into the contribution of individual features to the model’s predictions. Specifically, features associated with a larger LA size (such as the LA’s area or minor axis length) negatively impact predictions (i.e. are associated with an increased predicted risk). Conversely, features associated with a smaller LV size show an opposite trend. Notably, the sphericity of the LA and LV cavity (defined as the ratio between their minor and the major axes) also show opposite trends. This is further confirmed by univariate analyses, presented in Supplementary Figure S6, showing that increased LA size and reduced LV size are associated with a higher risk of disease progression.

#### 4.3.2 External validation

When evaluated externally in the Spanish Cohort, the model demonstrated strong transferability, reaching a C-index of 0.67 (95% CI [0.46, 0.88]). The corresponding lift curve is presented in Supplementary Figure S2.

Formal calibration assessment at 3 years was limited by the small number of events (n=6) in the external validation cohort, resulting in wide confidence intervals for the E/O ratio (1.04, 95% CI [0.57−3.13]), preventing definitive conclusions regarding calibration. Calibration plots stratified by tertiles of predicted risk (Supplementary Figure S8) showed general agreement between predicted and observed 3-year event probabilities, although these findings should be interpreted with caution given the limited sample size.

### 4.4 Area-derived LACI performance evaluation

As increased LA size and reduced LV size were associated with an increased risk of disease progression (Figure 2c and Supplementary Figure S6) area-derived LACI was considered as a simple and actionable echocardiographic marker for further analyses. Area-derived LACI is defined as the ratio between the LA and LV areas (measured at ED from apical 4-chamber views). The median [IQR] area-derived LACI was 0.40[0.32,0.54] in the discovery cohort and 0.47[0.37−0.57] in the validation cohort. Using this criterion as a risk score, its prognostic performance was assessed in both the discovery and validation cohorts.

#### 4.4.1 Area-derived LACI performance on discovery and validation cohort

**Figure 3:**
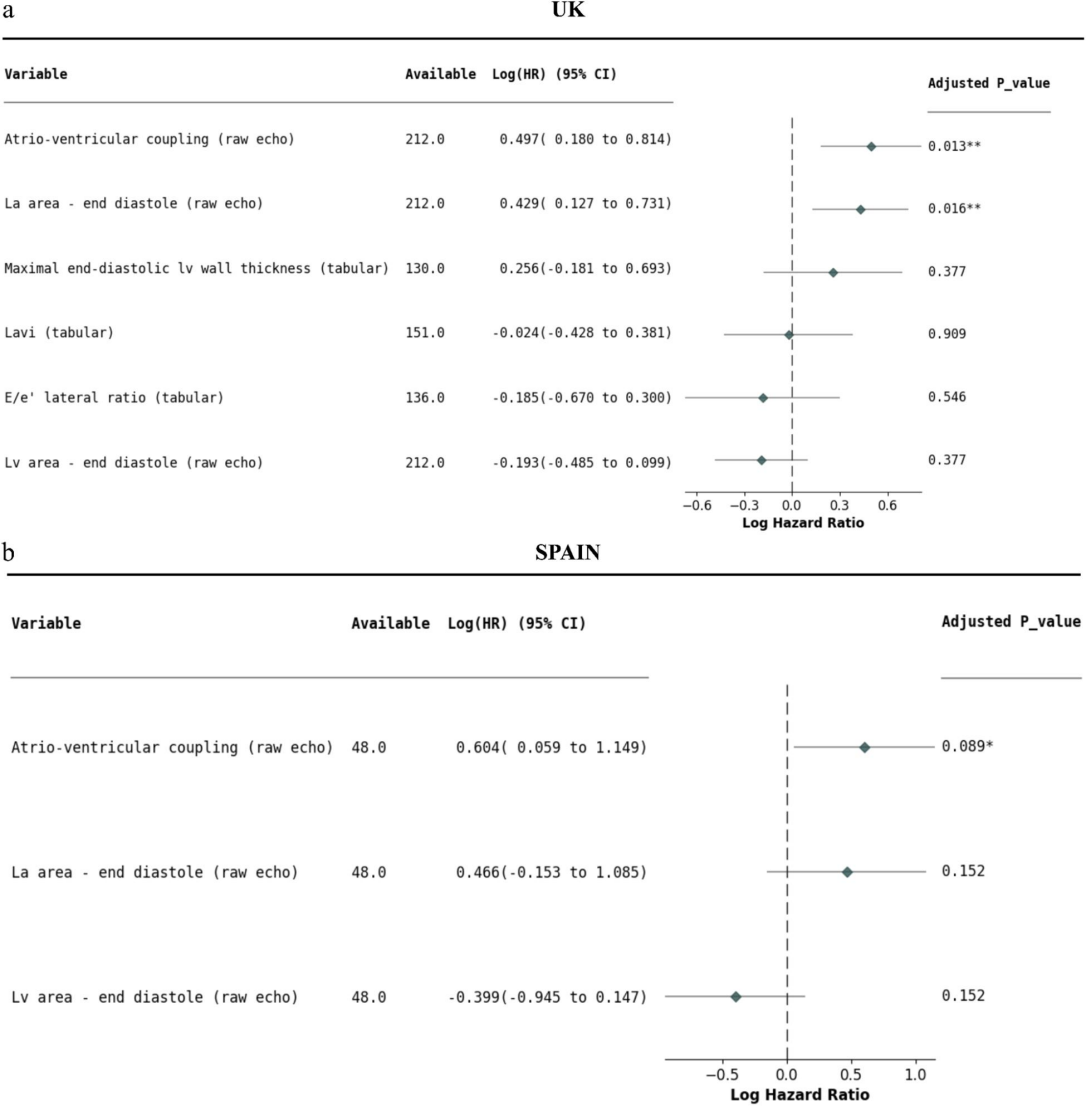
Forest plot of univariate analysis results in both cohorts. The plot displays the log Hazard Ratio (log(HR)) with 95% Confidence Intervals (95% CI), the number of observations (Available), and the adjusted *p*-values using the Benjamini-Hochberg correction (Adjusted *P*_value). The analysis is adjusted for age. **(a)** UK (discovery cohort). **(b)** Spain (validation cohort). LV, left ventricle; LA, left atrium; LAVi, left atrial volume index.

The criteria used as a risk score reached a C-index of 0.67 (95% CI [0.58, 0.77]) in the discovery cohort. Results for the univariate analysis compared to baseline variables are displayed in Figure 3a. In univariate Cox analysis, area-derived LACI was significantly associated with disease progression (HR per unit increase: 1.64, 95% CI: [1.20−2.26], adjusted p=0.013, equivalent to HR=1.05, 95% CI: [1.02--1.09] per 0.1-unit increase). The effect of area-derived LACI (measured at ED from apical 4-chamber views) is stronger, with higher statistical significance than for instance LA area or LV area alone. It shows that the prognostic signal lies in the coupling of the LA and LV geometries. Second, area-derived LACI as a risk score outperforms other known echocardiographic parameters (measured by clinician) such as LAVi, maximal end-diastolic LV wall thickness or E/e’ ratio. After adjustment for age and correction for multiple testing, area-derived LACI remains the only statistically significant predictor of disease progression. Finally, univariate analyses presented in Supplementary Figure S6 show that area-derived LACI’s prognostic capacity outperforms that of all other shape radiomic features used in the ML model.

In the external validation cohort, area-derived LACI reached a C-index of 0.72 (95% CI [0.56, 0.88]). Results from the univariate analysis, shown in Figure 3b, show that area-derived LACI remained associated with disease progression (HR per unit increase: 1.83, 95% CI: 1.06--3.16, adjusted p=0.089, equivalent to HR=1.06, 95% CI: [1.01--1.12] per 0.1-unit increase). They follow the same trend as those observed in the UK center, with the coupling of LA and LV having a stronger effect, and higher statistical significance, than LA and LV alone.

#### 4.4.2 Area-derived LACI, an echocardiographic marker to discriminate high-risk patients

The area-derived LACI criteria is then used to stratify patients according to their risk of disease progression (as described in Section 2.2.4).

**Figure 4:**
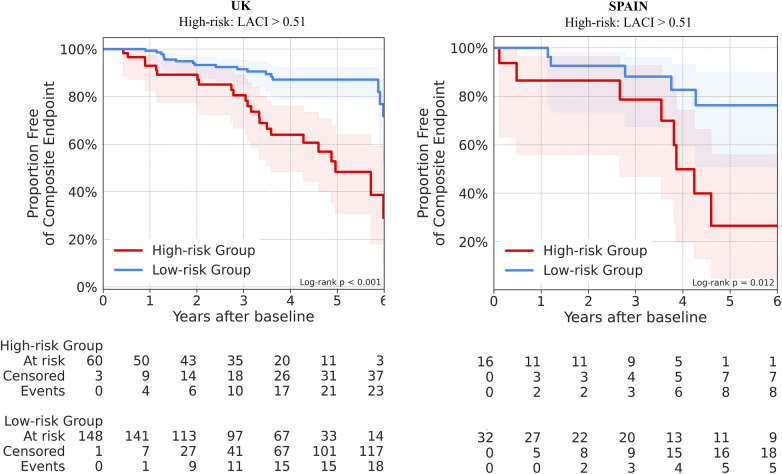
Kaplan-Meier estimates for the populations classified as high and low risk by a threshold established to identify patients who were the 30% more at risk in the discovery cohort (area-derived LACI > 0.51) applied to the discovery cohort (left) and the validation cohort (right). The x-axis represents the years following the baseline echocardiography exam, while the y-axis represents the progression-free composite endpoint population. The high-risk group in the validation cohort accounts for 33% of the overall population. (Log-rank p<0.001 in the discovery cohort; log-rank p=0.012 in the validation cohort).

Kaplan-Meier (KM) estimates and corresponding *p*-values (log-rank test) confirmed the robustness of the echocardiographic marker in stratifying the population. The stratification was based on the top 30% of area-derived LACI values in the UK cohort, chosen to match the incidence rate observed in the SOLENOID study. This corresponded to a threshold of area-derived LACI > 0.51. This threshold was then applied to the Spanish cohort. As illustrated in Figure 4, the differences in survival distributions were statistically significant ($p<10^{-5}$ in the UK center; p=0.013 in the Spanish cohort). The high-risk group in the discovery cohort, with area-derived LACI > 0.51, had a 3-year risk of disease progression of 19% (95% CI [13%, 36%]) compared to 8% (95% CI [5%, 15%]) for the low-risk group area-derived LACI ≤0.51. The high-risk group in the validation cohort with area-derived LACI >0.51 had a 3-year risk of disease progression of 21% compared to 12% for the low-risk group area-derived LACI ≤ 0.51.

Additional analyses (presented in Supplementary Figures S3, S4) using thresholds adjusted by ±5% demonstrated consistent results across all thresholds and cohorts. The 15% cutoff (LACI threshold = 0.65) also remained significant in the discovery cohort. In the validation cohort, the high-risk group showed a higher 3-year event rate, but statistical significance was not reached, likely reflecting limited sample size (Supplementary Figure S5).

## 5 Discussion

Assessing disease trajectories in HCM patients remains challenging, especially in pre-symptomatic patients where simple markers for disease progression are lacking. In the present study, by leveraging imaging data from the SOLENOID registry, a large cohort of pre-symptomatic patients (15), a machine learning model for disease progression was developed, highlighting several predictive features. In view of promoting its applicability in daily life, a simple and actionable criteria was identified, the left atrioventricular coupling index (area-derived LACI), which was evaluated and validated in an external cohort.

Although most HCM patients remain asymptomatic, a substantial proportion will develop cardiac events, mainly AF, SCD and HF (27). Assessing patient’s trajectories in HCM is still largely unresolved and the existing data are heavily influenced by the endpoint being assessed. Currently, available risk evaluation based on conventional clinical and imaging variables can stratify the 5-year SCD risk, or the development of de novo AF (28, 29). However, most models do not predict long-term progression to HF especially for pre-symptomatic patients. Only one model has been developed to predict the 5-year risk of developing several HF outcomes in symptomatic HCM from a single institution. Seventeen clinical and imaging variables were included and allow an overall good accuracy (C-index of 0.81), but the study lacks external validation (14). Thus, new models focused on pre-symptomatic patients and with external validation are needed to improve risk stratification of HF. Advanced imaging markers in addition to conventional risk factors could help refining risk stratification in HCM, as suggested by a recent meta-analysis (7).

To the best of our knowledge, this is the first study to propose such a (ML) model that leverages raw images to predict disease progression in the context of pre-symptomatic HCM. We emphasize that our pipeline, starting from raw apical 4-chamber sequences, requires no manual intervention or correction, aiming at reducing inter operational variability. The final ML model achieves consistent performance between the discovery and external validation cohorts (C-index of 0.66 (95% CI: [0.54, 0.77]) and 0.67 (95% CI: 0.46, 0.88]) respectively), showing overall stability of the pipeline across datasets, although the wider interval in the validation cohort reflects its limited sample size. In the external validation cohort, the model shows no evidence of systematic miscalibration at 3 years, although estimates are imprecise due to the small sample size. Interestingly, by using an agnostic approach, the main features that contribute to the model are related to the left atrium (increased LA size) and left ventricle morphology (reduced LV size), which is in line with the literature regarding patterns of disease progression for HCM (8–11, 13), even though this study focuses on pre-symptomatic patients.

Several echocardiographic parameters have been associated separately with major cardiac events in symptomatic HCM but few with disease progression (7). More importantly, their impact is highly variable depending on the model and the outcomes. In HCM patients, LA dysfunction is commonly observed even with preserved LV systolic function, due to multiple causes. While LA and LV parameters have been associated with various CV outcomes separately, some reports have suggested that the coupling between the LA and LV in the heart could be used as an independent echocardiographic marker for risk stratification. While originally derived (30) as the ratio of the volume of the left atrium and that of the left ventricle from CMR imaging, LACI has been investigated in computed tomography (31) or echocardiography, for various populations and diseases (32–34). In the MESA cohort, including patients without history of CV diseases, LACI was associated with cardiac events such as HF, AF, death (30). Similar trends were observed in HFpEF patients who, like HCM patients present diastolic dysfunction (35). Only one study has investigated LACI in an HCM-specific population (all NYHA functional classes), showing that LACI was a prognostic marker for the risk of new-onset of AF (11).

We offer a simple and actionable echocardiographic marker to identify patients at risk of progression among pre-symptomatic HCM patients. Estimating LACI using the areas from a single apical 4-chamber view only was sufficient to predict disease progression and stratify patients, without the need to estimate the LA and LV volumes (36). Besides, area-derived LACI was derived from echocardiography rather than CMR imaging and therefore is more suited to routine clinical practice. Therefore, one might consider using area-derived LACI in a clinical trial setting, to select patients at high-risk of events that are most likely to benefit from a therapeutic intervention. Moreover, the 0.51 area-derived LACI threshold used to define high-risk patients is consistent with values reported in prior studies (11). By contrast, the DERIVATE registry (37) reported a lower CMR-derived LACI threshold (21%) in patients with HFrEF. The main reason for this discrepancy is the definition of LACI itself: volumetric in DERIVATE vs. area-derived in our study. Since areas and volumes scale differently with chamber size, these two metrics are expected to produce different numerical thresholds. This geometric distinction likely explains a major part of the discrepancy, with additional contributions from patient populations, cardiac geometry, and endpoint definitions. Whether these findings could translate in a more advanced population (Functional NYHA II+) was not addressed in the present study.

### 5.1 Study limitations

There are several important limitations to this analysis. First, it relies on retrospective data and was not prespecified within the original SOLENOID protocol, making it susceptible to selection or inclusion biases and risk of missing data, and should therefore be considered exploratory. Several clinical and echocardiographic variables were missing for some patients, and atrial fibrillation status at the time of echocardiographic acquisition was not systematically recorded, preventing assessment of its potential impact on measurement reliability.

The study focuses on two European cohorts (from UK and Spain) and therefore may not be generalizable to other populations such as patients from North America or Asia. The external validation cohort was relatively small (N=48 patients), which limited the precision of the calibration and may affect the stability of the reported C-index estimates and the generalizability of the proposed LACI threshold. Results would benefit from confirmation in larger cohorts with sufficient statistical power.

CMR data, including late gadolinium enhancement and fibrosis burden, were not available in this study, preventing comparison of area-derived LACI with established CMR-based prognostic markers. Direct comparison with established HCM risk scores was also not possible due to missing variables in this retrospective analysis. Other interesting dynamic parameters such as velocities or strain were not included in our model, although they were included in the univariate analysis when available).

Area-derived LACI was based on areas rather than volumes making the comparison with literature more difficult, although its simplicity makes it more suitable for clinical practice. In addition, the proposed LACI threshold was derived specifically within the automated pipeline and may require recalibration before application to manually measured echocardiographic parameters.

The C-index values of 0.66--0.67 reflect a modest discriminative performance of the ML model, which may limit its standalone clinical applicability even though the derived area-based LACI showed improved prognostic value.

Finally, the threshold to identify patients at risk of event (30%) was selected based on the epidemiological findings of the SOLENOID registry.

## 6 Conclusion

This study shows that echocardiography, combined to a fully automated ML pipeline, can contribute to the early assessment of disease progression and risk stratification in pre-symptomatic HCM patients. We identified the left atrioventricular coupling index at end diastole, derived from our model, as an actionable feature that improved risk stratification compared to the conventional LA size alone. Our findings provide new insights into the natural history of early-stage HCM patients and may help to better manage HCM patients at an early stage.

## Data Availability

The data analyzed in this study is subject to the following licenses/restrictions: the datasets analyzed for this study are not publicly available due to ethical and privacy restrictions. Requests to access the raw data should be directed to the corresponding author and are subject to third-party approval. Requests to access these datasets should be directed to Antoine Olivier, antoine.olivier@owkin.com.
