# Supplementary material for: A fully automated machine learning assisted pipeline to predict disease progression for early-stage hypertrophic cardiomyopathy from echocardiography
Source: Front Cardiovasc Med. 2026 Aug 3;13:1875305. doi: 10.3389/fcvm.2026.1875305 (PMC13477913; doi:10.3389/fcvm.2026.1875305)
Supplement: Supplementary file 1 [file Datasheet1.pdf]

Supplementary Material

1 SUPPLEMENTARY METHODS

1.1 Segmentation model and end-diastole identification

In the initial step of the pipeline, an automated segmentation of cardiac structures was performed using a supervised Deep Learning segmentation model based on the U-net architecture. The model was trained using the publicly available "Cardiac Acquisitions for Multi-structure Ultrasound Segmentation" (CAMUS) dataset. This model takes as input a B-mode apical 4-chamber view and outputs masks that identify the three main structures on the left side of the heart: the myocardium, left ventricle and left atrium. In the training procedure, the data was split with a 5-fold cross-validation, resulting in five models. For each split, performance was monitored by evaluating the Dice and Hausdorff metric on the corresponding validation fold, and the final model was obtained by averaging the predictions of the five individual models. For each sequence, this model was applied to all frames. The temporal evolution of the left ventricle area was then used to automatically identify an end-diastole (ED) frame for each patient, by selecting the maximum measured LV area across the sequence.

The segmentation model achieved on the CAMUS dataset a per-channel Dice score of 0.947 ± 0.004 for the left ventricle, 0.879 ± 0.008 for the myocardium, and 0.928 ± 0.007 for the left atrium, with corresponding Hausdorff distances of 7.5 ± 0.4 mm, 9.3 ± 0.4 mm, and 8.3 ± 0.5 mm respectively, indicating good spatial agreement between predicted masks and expert annotations.

An automated two-stage quality control procedure was applied to ensure segmentation reliability. First, each segmented frame was assessed independently: the predicted masks for the left ventricle, myocardium, and left atrium were required to exceed minimum area thresholds and to form a single connected component, in order to exclude degenerate or fragmented segmentations. Frames failing either criterion were flagged as invalid. Then, a sequence was kept only if fewer than 30% of frames were flagged as invalid. At the patient level, subjects were excluded if all available echocardiographic sequences from their exam failed this criterion. Overall, 5% were excluded due to insufficient segmentation quality.

1.2 Feature extraction

For each patient, handcrafted parameters, known as "radiomics", were extracted for each structure of the ED frame. These radiomics encompass a set of statistical characteristics, including first-order descriptors that capture information about pixel intensity histograms, second-order descriptors that depict texture, and shape descriptors. To address dimensionality concerns and prioritize the use of reproducible and interpretable features, the analysis was exclusively focused on shape radiomic features. They included elongation, major and minor axis length, maximum diameter, mesh and pixel surface, perimeter, perimeter to surface ratio, and sphericity.

1.3 Model training and validation

To capture potential non-linear relationships and feature interactions in predicting disease progression in early-stage HCM patients from extracted radiomics, a supervised survival analysis ML approach was applied. Specifically, Gradient Boosting Trees algorithms were used, optimized to maximize the log likelihood for the Accelerated Failure Time (AFT). Unlike Cox models, AFT models do not rely on the

proportional hazards condition. The implementation was based on the end-to-end tree boosting system, XGBoost.

The performance on the discovery cohort used as a training set was evaluated by selecting optimal models and corresponding hyperparameters (controlling for the tree complexity and regularization), using a nested cross-validation. Hyperparameter optimization focused on maximizing the concordance index (C-index) within each inner loop. In addition to the C-index, the lift curve was computed to assess the model's ability to stratify risk. The lift curve offers insight into the model's performance in identifying individuals with an enriched event rate at four years, making it a valuable complementary metric for evaluating risk prediction.

The final model was obtained applying the hyperparameter optimization pipeline on the full discovery cohort. To evaluate its external validity, the model was subsequently tested on the validation cohort. Performance metrics for this external validation cohort were similarly evaluated using the C-index and lift curve. To ensure robust estimates, bootstrapping techniques were employed ($n = 100$) to generate confidence intervals for both the lift curve and the C-index. This analysis provided valuable insights into the model's generalizability beyond the original training dataset and its performance consistency in a new patient population.

Software and Tools

Software and libraries

Analyses were performed using algorithms written in Python 3.11, along with the following external libraries: NumPy (1.26.4), lifelines (0.27.8), Optuna (3.4.0), pandas (1.5.3), pyradiomics (3.0.1), scikit-learn (1.5.2) and XGBoost (1.7.6).

Pyradiomics shape features

The following 2D shape radiomic features were extracted using the open-source pyradiomics library at end-diastole (ED) for each of the three segmented cardiac structures (myocardium, left ventricle, and left atrium):

- original_shape2D_Elongation
- original_shape2D_MajorAxisLength
- original_shape2D_MaximumDiameter
- original_shape2D_MeshSurface
- original_shape2D_MinorAxisLength
- original_shape2D_Perimeter
- original_shape2D_PerimeterSurfaceRatio
- original_shape2D_PixelSurface
- original_shape2D_Sphericity

Optuna hyperparameter optimization

Hyperparameter optimization was performed using Optuna with 100 trials. The best hyperparameters selected for the final model are reported in Supplementary Table 1.

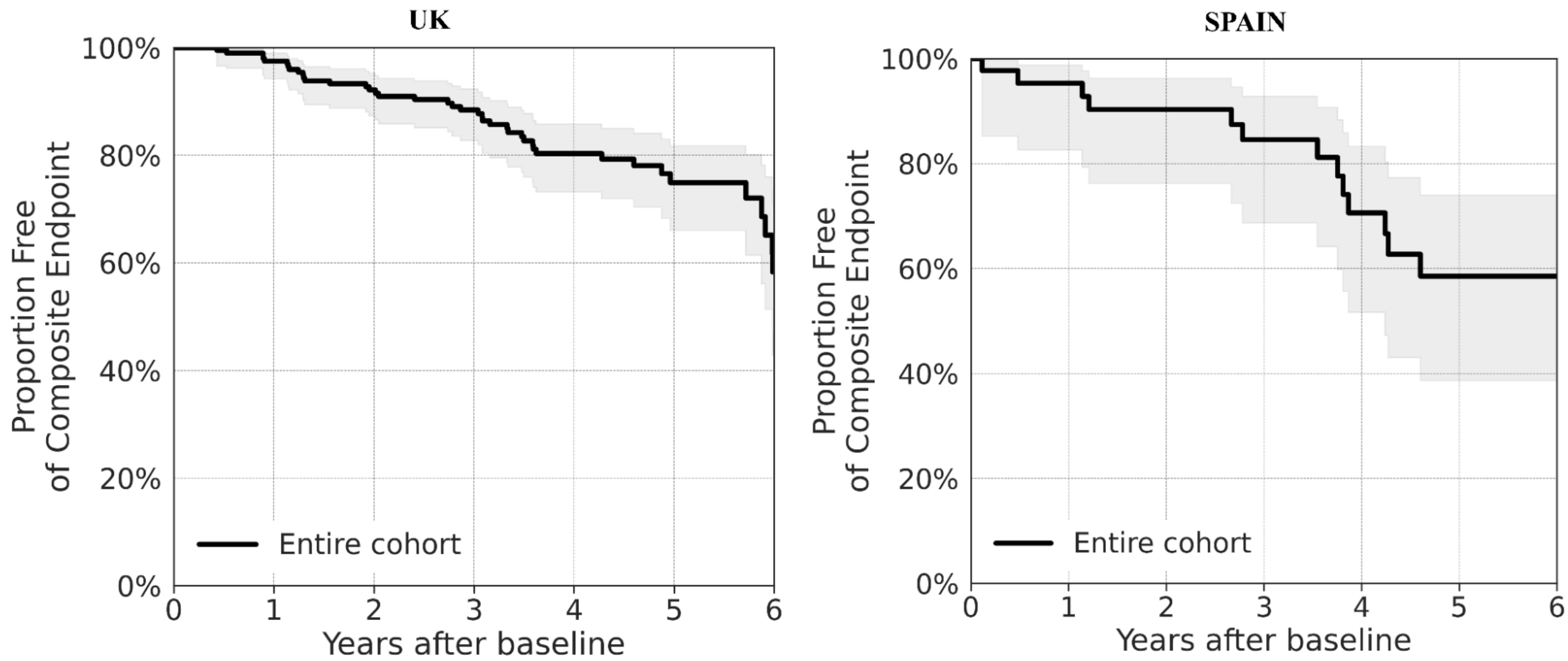

Figure S1. Survival curves in the discovery and validation cohort. Kaplan-Meier curves for the discovery cohort (left) and the validation cohort (right). The x-axis represents the years following the baseline echocardiography exam, while the y-axis represents the progression-free composite endpoint population.

2 SUPPLEMENTARY TABLES AND FIGURES

**Hyperparameter**	**Value**
`learning_rate`	0.031
`max_depth`	3
`num_boost_round`	87
`min_child_weight`	8
`gamma`	4.76
`subsample`	0.821
`colsample_bytree`	0.705
`lambda`	0.434
`alpha`	0.521
`aft_loss_distribution_scale`	1.42

Table S1. Best hyperparameters of the XGBoost survival model selected via Optuna.

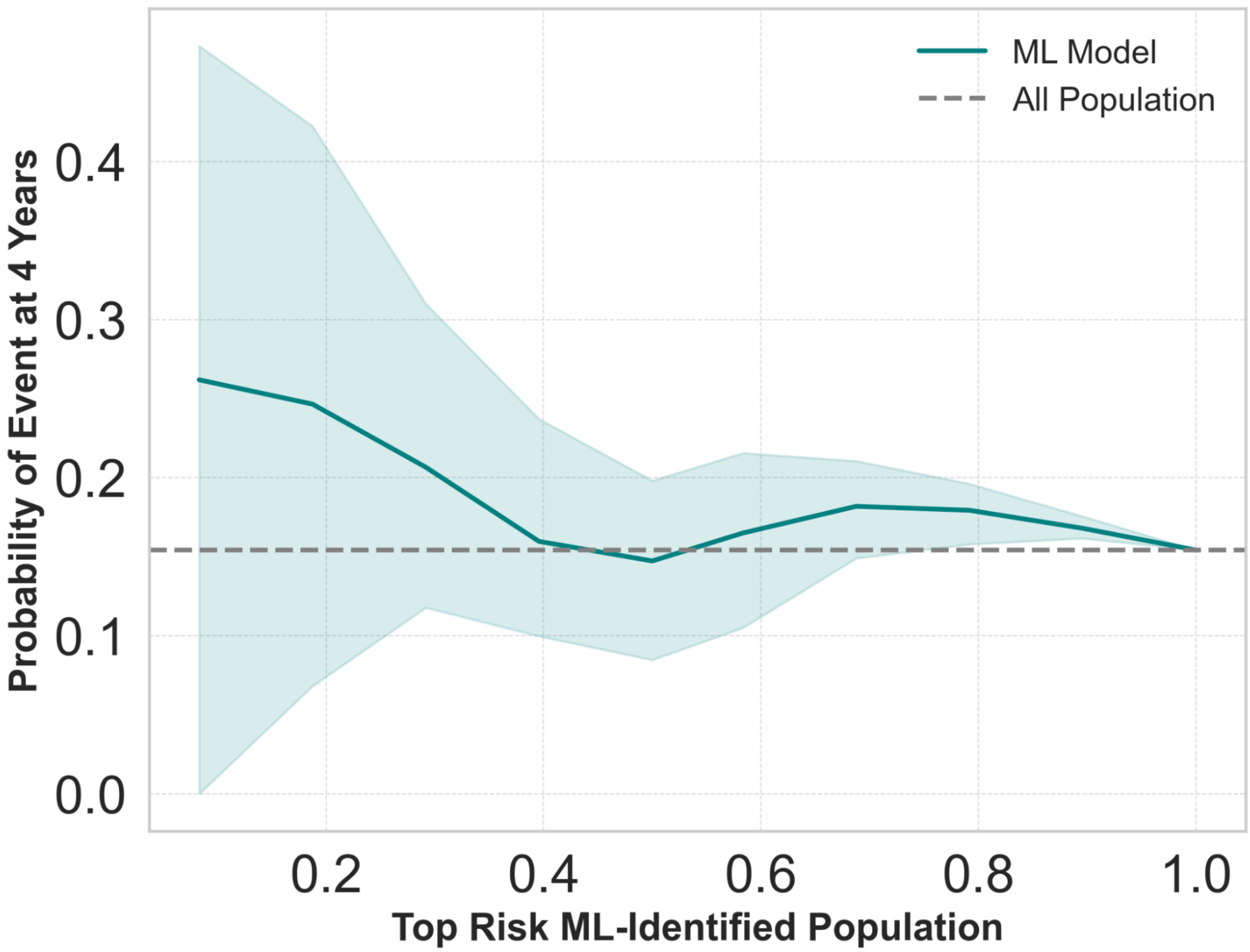

Figure S2. External Validation of the prognostic model. Lift curve at 4 years for the trained prognostic model evaluated on the validation cohort. Following a bootstrap ($n = 100$), the main curve is derived from the average across all curves, while the confidence intervals are based on the 0.1 and 0.9 quantiles of the predicted values for the validation cohort. Abbreviations: ML Machine learning

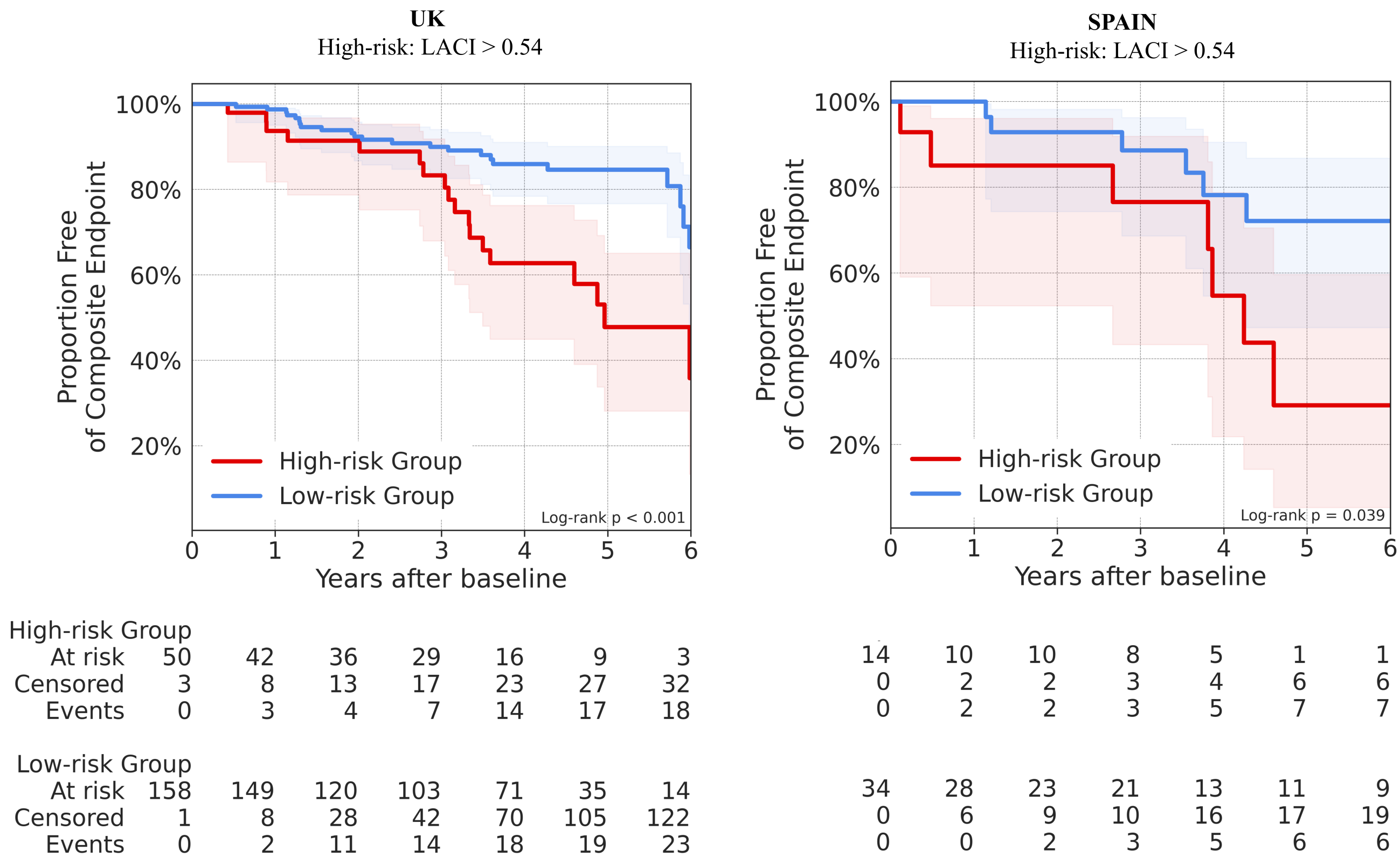

Figure S3. Proportion free of composite endpoint for the populations classified as high and low risk by a threshold established to identify patients who were 25% more at risk in the discovery cohort (area-derived LACI > 0.54) applied to the discovery cohort (left) and the validation cohort (right). The x-axis represents the years following the echocardiography exam, while the y-axis represents the progression-free composite endpoint population. The high-risk group in the validation cohort accounts for 29% of the overall population. (Log-rank $p < 0.001$ in the discovery cohort; log-rank $p = 0.039$ in the validation cohort).

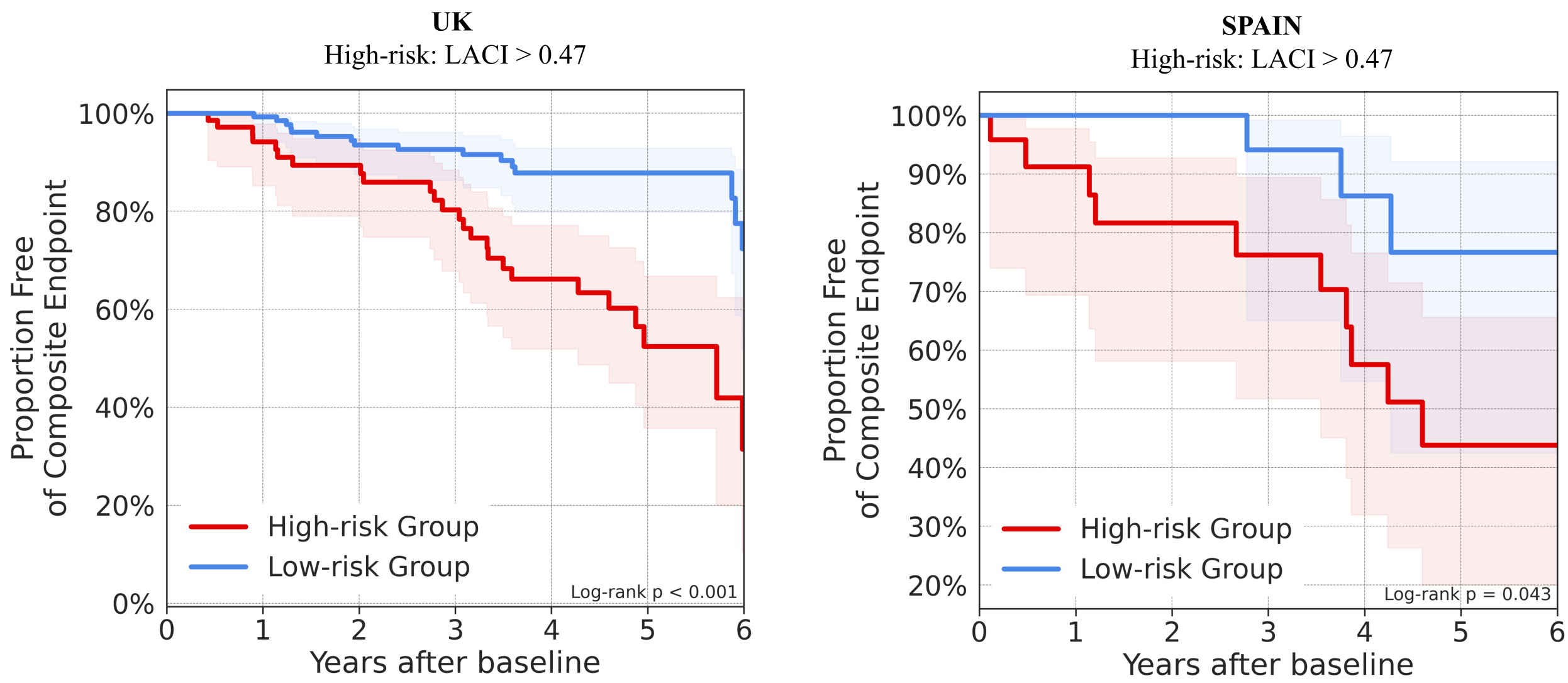

UK

	0	1	2	3	4	5	6
**High-risk Group**							
At risk	71	61	52	42	25	13	3
Censored	3	9	15	20	30	38	46
Events	0	4	7	12	19	23	25
**Low-risk Group**							
At risk	137	130	104	90	62	31	14
Censored	1	7	26	39	63	94	108
Events	0	1	8	9	13	13	16

SPAIN

	0	1	2	3	4	5	6
**High-risk Group**							
At risk	24	19	15	13	9	4	4
Censored	0	3	5	6	7	10	10
Events	0	2	4	5	8	10	10
**Low-risk Group**							
At risk	24	19	18	16	9	8	6
Censored	0	5	6	7	13	13	15
Events	0	0	0	1	2	3	3

Figure S4. Proportion free of composite endpoint for the populations classified as high and low risk by a threshold established to identify patients who were 35% more at risk in the discovery cohort (area-derived LACI > 0.47) applied to the discovery cohort (left) and the validation cohort (right). The x-axis represents the years following the echocardiography exam, while the y-axis represents the progression-free composite endpoint population. The high-risk group in the validation cohort accounts for 50% of the overall population. (Log-rank $p < 0.001$ in the discovery cohort; log-rank $p = 0.043$ in the validation cohort).

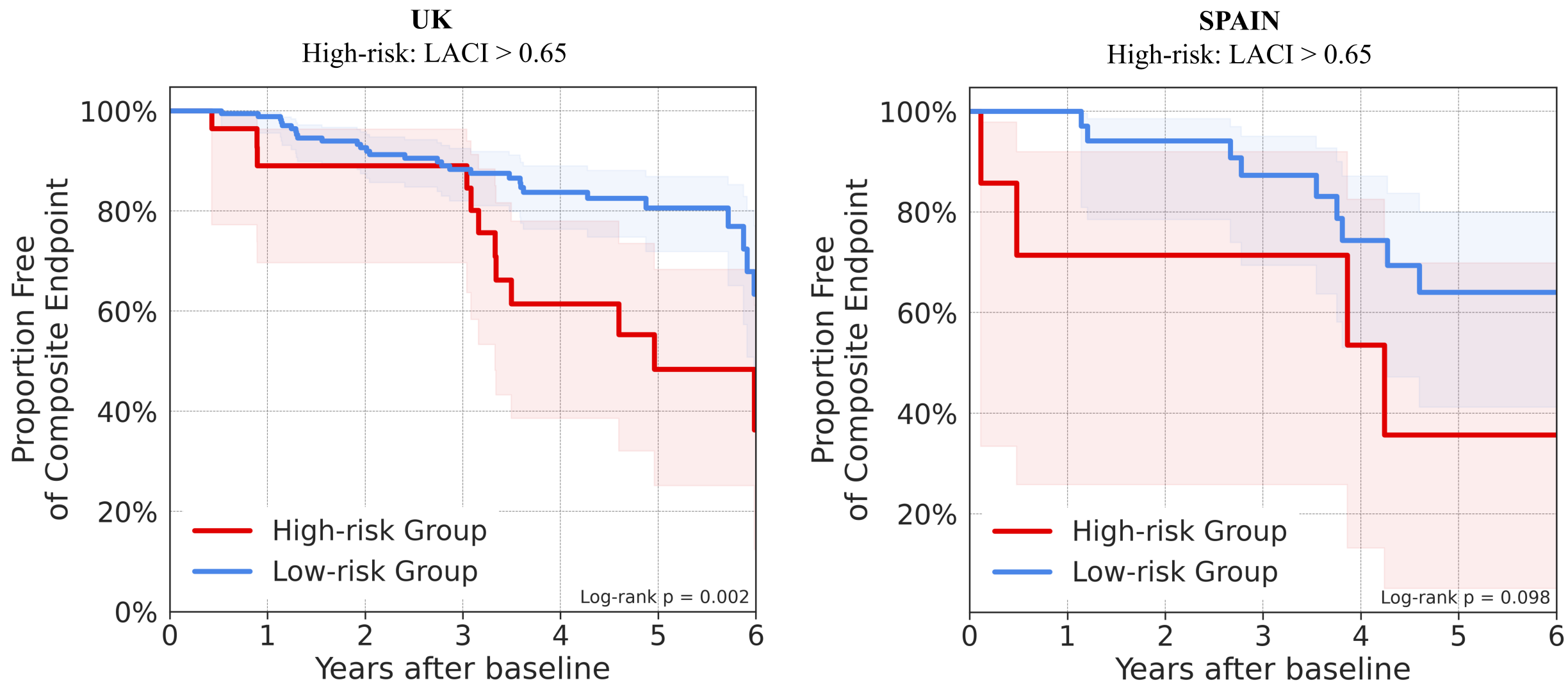

High-risk Group	UK 0	1	2	3	4	5	6	SPAIN 0	1	2	3	4	5	6
At risk	28	24	21	20	11	7	3	7	4	4	4	3	1	1
Censored	3	4	7	8	11	13	16	0	1	1	1	1	2	2
Events	0	3	3	3	9	11	12	0	2	2	2	3	4	4

Low-risk Group	UK 0	1	2	3	4	5	6	SPAIN 0	1	2	3	4	5	6
At risk	180	167	135	112	76	37	14	41	34	29	25	15	11	9
Censored	1	12	34	51	82	119	138	0	7	10	12	19	21	23
Events	0	2	12	18	23	25	29	0	0	2	4	7	9	9

Figure S5. Proportion free of composite endpoint for the populations classified as high and low risk by a threshold established to identify patients who were 15% more at risk in the discovery cohort (area-derived LACI > 0.65) applied to the discovery cohort (left) and the validation cohort (right). The x-axis represents the years following the echocardiography exam, while the y-axis represents the progression-free composite endpoint population. The high-risk group in the validation cohort accounts for 15% of the overall population. (Log-rank $p = 0.002$ in the discovery cohort; log-rank $p = 0.098$ in the validation cohort).

Variable	Available	Log(HR) (95% CI)	Adjusted P_value
**LACI**			
Atrio-ventricular coupling (raw echo)	212.0	0.497(0.180 to 0.814)	0.029**
**All radiomic features**			
Original_shape2d_minoraxislength_la_ed	212.0	0.529(0.207 to 0.851)	0.029**
Original_shape2d_meshsurface_la_ed	212.0	0.429(0.127 to 0.731)	0.03**
La area - end diastole (raw echo)	212.0	0.429(0.127 to 0.731)	0.03**
Original_shape2d_perimeter_la_ed	212.0	0.365(0.069 to 0.661)	0.073*
Original_shape2d_majoraxislength_la_ed	212.0	0.319(0.035 to 0.604)	0.111
Original_shape2d_maximumdiameter_la_ed	212.0	0.310(0.025 to 0.596)	0.116
Original_shape2d_sphericity_la_ed	212.0	0.302(0.010 to 0.595)	0.134
Original_shape2d_sphericity_myo_ed	212.0	0.283(-0.028 to 0.593)	0.208
Original_shape2d_elongation_la_ed	212.0	0.201(-0.119 to 0.521)	0.361
Original_shape2d_perimetersurfaceratio_lv_ed	212.0	0.200(-0.084 to 0.485)	0.359
Original_shape2d_elongation_myo_ed	212.0	0.131(-0.192 to 0.455)	0.52
Original_shape2d_elongation_lv_ed	212.0	0.031(-0.266 to 0.329)	0.836
Original_shape2d_pixelsurface_myo_ed	212.0	-0.066(-0.391 to 0.259)	0.718
Original_shape2d_meshsurface_myo_ed	212.0	-0.066(-0.391 to 0.259)	0.718
Original_shape2d_perimetersurfaceratio_myo_ed	212.0	-0.101(-0.437 to 0.235)	0.622
Original_shape2d_sphericity_lv_ed	212.0	-0.108(-0.410 to 0.194)	0.566
Original_shape2d_minoraxislength_lv_ed	212.0	-0.129(-0.421 to 0.162)	0.49
Original_shape2d_maximumdiameter_lv_ed	212.0	-0.138(-0.432 to 0.156)	0.475
Original_shape2d_perimeter_lv_ed	212.0	-0.158(-0.461 to 0.145)	0.43
Original_shape2d_majoraxislength_lv_ed	212.0	-0.168(-0.469 to 0.133)	0.404
Original_shape2d_minoraxislength_myo_ed	212.0	-0.170(-0.474 to 0.133)	0.404
Original_shape2d_meshsurface_lv_ed	212.0	-0.193(-0.485 to 0.099)	0.361
Lv area - end diastole (raw echo)	212.0	-0.193(-0.485 to 0.099)	0.361
Original_shape2d_maximumdiameter_myo_ed	212.0	-0.199(-0.507 to 0.109)	0.361
Original_shape2d_majoraxislength_myo_ed	212.0	-0.225(-0.528 to 0.077)	0.337
Original_shape2d_perimeter_myo_ed	212.0	-0.227(-0.532 to 0.078)	0.337
Original_shape2d_perimetersurfaceratio_la_ed	212.0	-0.491(-0.817 to -0.165)	0.029**

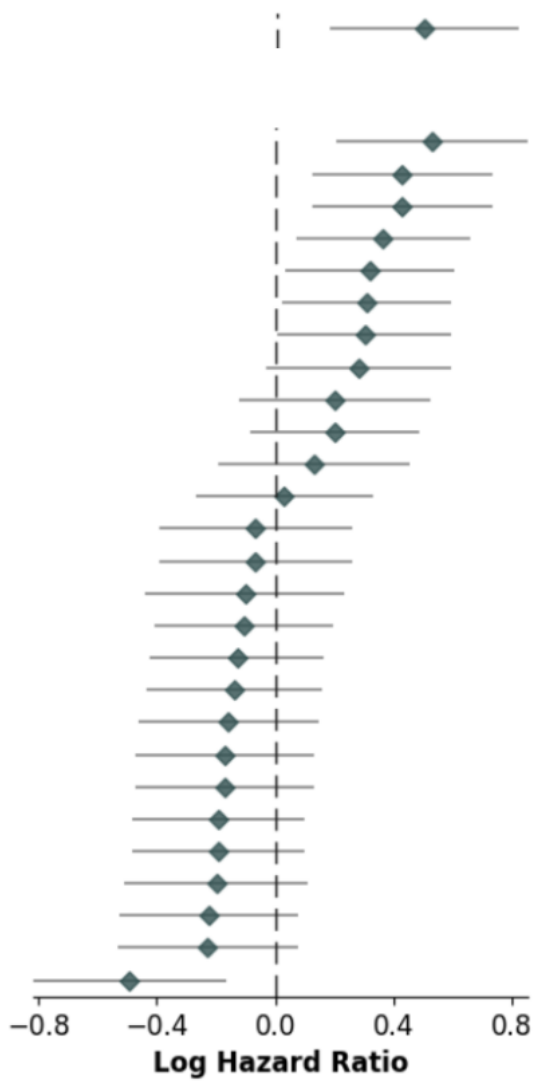

Figure S6. Forest plot of univariate analysis results for area-derived LACI and all shape radiomic features. The plot displays the log Hazard Ratio (log(HR)) with 95% Confidence Intervals (95% CI), the number of observations (Available), and the adjusted p-values using the Benjamini-Hochberg correction (Adjusted P_value). The analysis is adjusted for age. Abbreviations : LV : left ventricle; LA: Left atrium; MYO: myocardium.

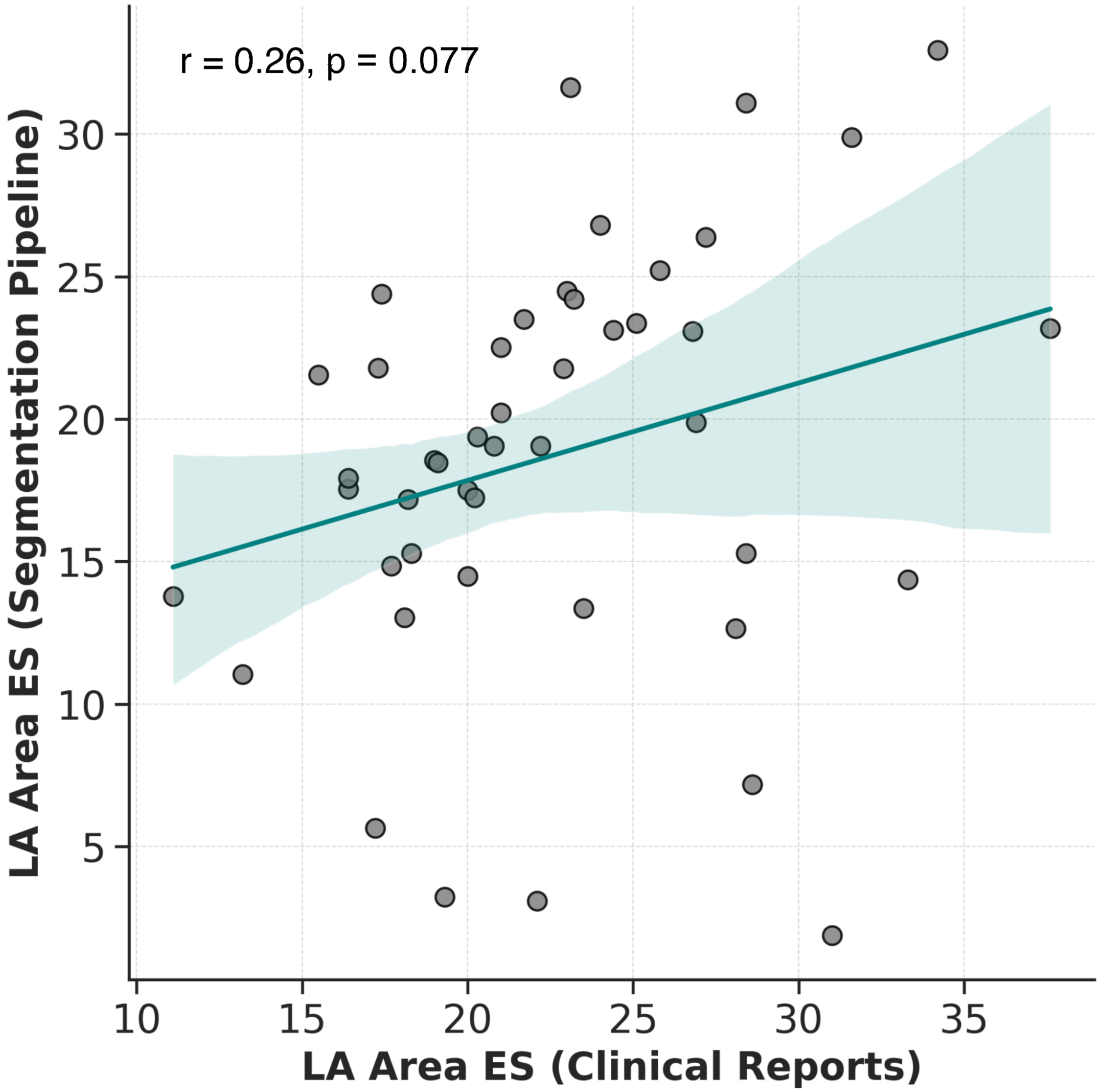

Figure S7. Correlation between automatically extracted and clinician-measured left atrial area. Pearson correlation between automatically extracted LA area at ES and clinician measurements for 46 patients (expressed in cm²) ($r = 0.26$, $p = 0.077$). Abbreviations: LA : left atrium; ES : end-systole

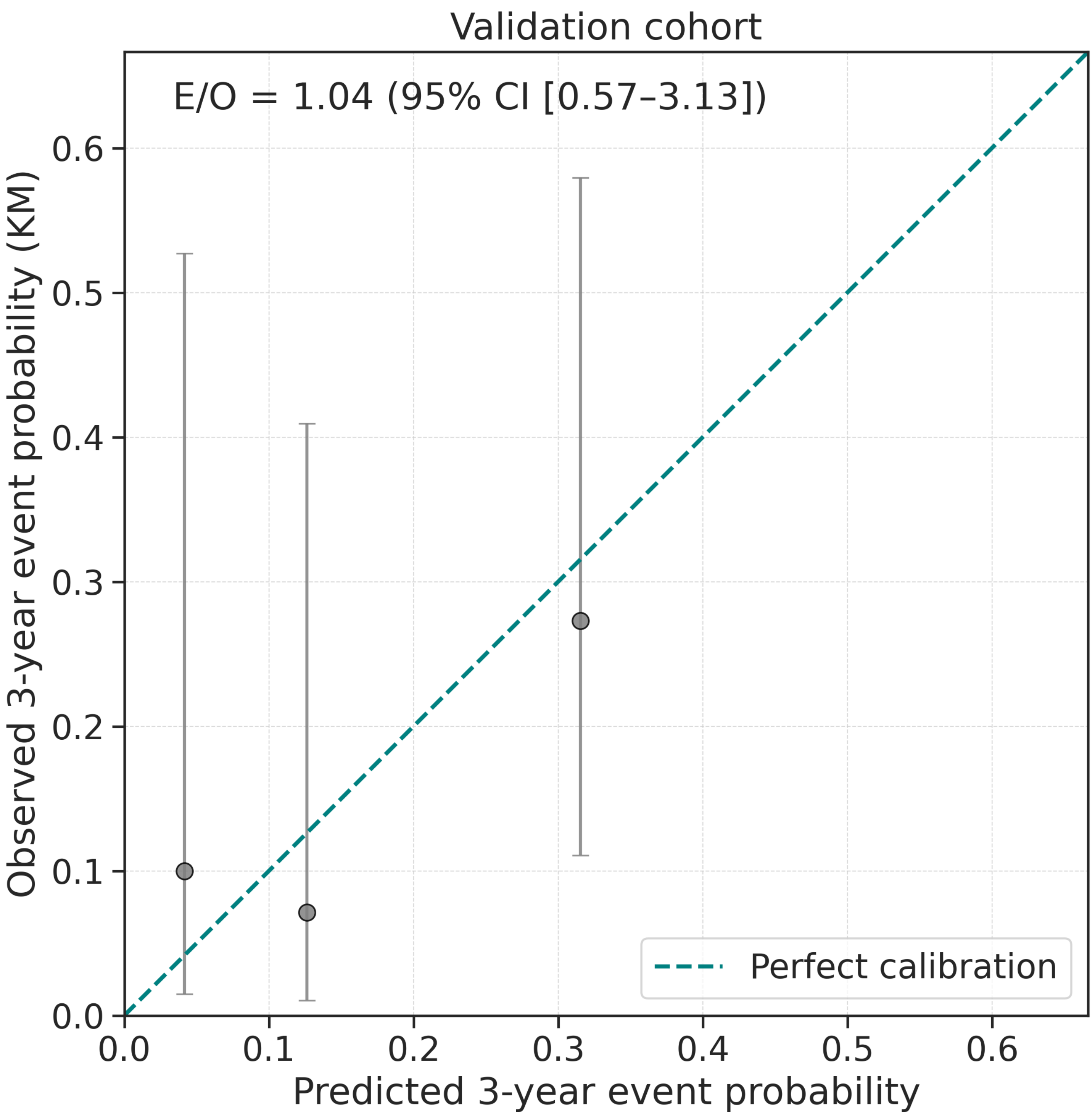

Figure S8. Comparison of predicted and observed 3-year event probabilities. Observed risks were estimated using Kaplan–Meier analysis at 3 years. Patients were grouped into tertiles of predicted risk to evaluate calibration across risk groups. The diagonal line represents perfect calibration between predicted and observed probabilities. Expected-to-observed (E/O) ratios with 95% CI were computed using bootstrap resampling ($n = 500$) on the log scale. Abbreviations: E/O: Expected/Observed ratio; KM: Kaplan–Meier; CI: confidence interval.
